# Gray-White Matter Ratio at the Level of the Basal Ganglia as a Predictor of Neurologic Outcomes in Cardiac Arrest Survivors: A Literature Review

**DOI:** 10.3389/fmed.2022.847089

**Published:** 2022-03-15

**Authors:** Fating Zhou, Hongxia Wang, Mengyao Jian, Zhiyuan Wang, Yarong He, Haizhen Duan, Lu Gan, Yu Cao

**Affiliations:** ^1^Department of Emergency Medicine, West China Hospital, Sichuan University, Chengdu, China; ^2^Laboratory of Emergency Medicine, West China Hospital, Sichuan University, Chengdu, China

**Keywords:** cardiac arrest (ca), cardiopulmonary resuscitation (cpr), neurologic outcome, gray-white matter ratio, basal ganglia (bg)

## Abstract

Loss of gray-white matter discrimination is the primary early imaging finding within of cranial computed tomography in cardiac arrest survivors, and this has been also regarded as a novel predictor for evaluating neurologic outcome. As displayed clearly on computed tomography and based on sensitivity to hypoxia, the gray-white matter ratio at basal ganglia (GWR-BG) region was frequently detected to assess the neurologic outcome by several studies. The specificity of GWR-BG is 72.4 to 100%, while the sensitivity is significantly different. Herein we review the mechanisms mediating cerebral edema following cardiac arrest, demonstrate the determination procedures with respect to GWR-BG, summarize the related researches regarding GWR-BG in predicting neurologic outcomes within cardiac arrest survivors, and discuss factors associated with predicting the accuracy of this methodology. Finally, we describe the effective measurements to increase the sensitivity of GWR-BG in predicting neurologic outcome.

## Introduction

Cardiac arrest (CA) is a commonly occurring and life-threatening event associated with high rate of mortality and disability. It has been reported that 45% of in-hospital and 30% of out-of-hospital CA patients are able to achieve return of spontaneous circulation (ROSC) with resuscitation efforts (1–3). However, most survivors have poor neurological outcome following hospital discharge due to hypoxic-ischemic brain injury sustained during CA (1, 4). Thus, accurately assessment neurological prognostication is essential to make appropriate therapeutic decisions in these patients.

At present, various examinations and tests, including clinical examinations, serum biomarker testing, electrophysiological tests, and neuroimaging are used to predict neurological outcome in adult comatose CA survivors (5). Among these methodologies, assessment of the gray-white matter ratio at the basal ganglia (GWR-BG) has been recommended as an effective tool for predicting neurologic outcome by several studies (6–8). GWR-BG is useful for predicting neurologic outcome, because the basal ganglia is sensitive to brain ischemia and hypoxia. Furthermore, gray matter and white matter in this region are clearly visible on cranial computed tomography (CT) (9). A previous retrospective analysis determined that the GWR was statistically significantly lower in comatose CA survivors as compared with normal controls (7). For those survivors, good neurological outcome patients presented with a higher GWR value than those poor in this study (7). Another study identified that GWR-BG values below 1.2 and 1.1 indicated severe neurological impairment and near-death status, respectively (10). Accumulating evidence has proven conclusively that GWR-BG is an early predicator of poor outcomes in CA survivors. However, the reported sensitivity varies significantly among reports. For example, a recent study reported that the sensitivity of GWR-BG in predicting poor outcomes in out-of-hospital CA was only 3.5% (11). However, another conducted study by Lee et al. found that the GWR-BG sensitivity for predicting poor outcomes in out-of-hospital CA survivors treated with extracorporeal membrane oxygenation (ECMO) was 88% (12). To identify factors that influence the sensitivity and prognostic accuracy of GWR-BG in predicting post-CA neurologic outcomes, we reviewed the molecular mechanisms of cerebral edema following CA herein.

## Pathophysiology of Cerebral Edema Following CA

**Figure 1:**
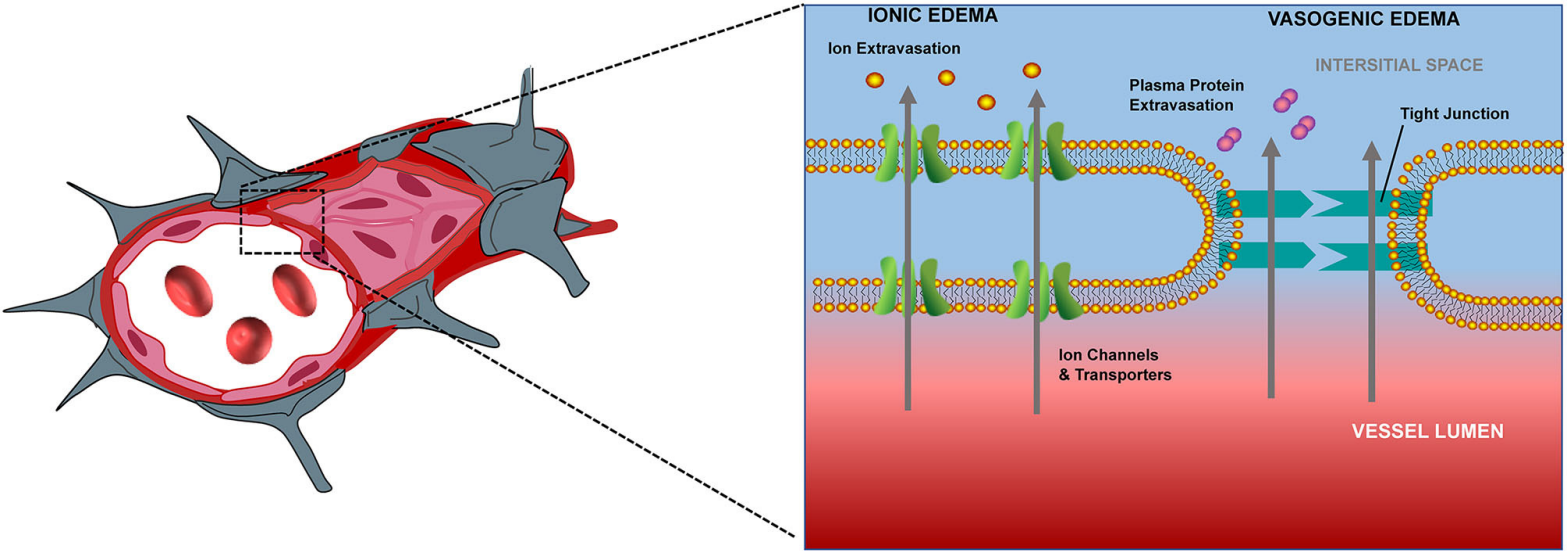
Schema of edema formation after cardiac arrest.

Adenosine triphosphate (ATP) production insufficiency following CA induces dysfunction of Na^+^-K^+^ ATPase pumps, eventually resulting in an influx of water into cells, and consequently, cerebral edema. Underlying processes mediating cerebral edema can be broadly categorized as ionic edema and vasogenic edema (Figure 1). Ionic edema is caused by intracellular osmotic imbalance and the consequent disruption of intracellular fluid homeostasis. Following cellular injury, excess ions and water accumulate in the extracellular compartment, thus establishing an osmotic gradient (9). This gradient drives the influx of ions and water into the cell, thereby causing tissue swelling. Unlike ionic edema, vasogenic edema is induced by damage to the blood-brain barrier. Disruption of the blood-brain barrier leads to increased permeability and albumin efflux into the surrounding vascular and interstitial space, thus inducing diffuse cerebral edema (9). Ionic (cellular) edema can occur within the first few minutes after circulation is ceased, while vasogenic edema appears hours later (13). During these processes, the tissue density of the cerebral gray matter (GM) decreases earlier in comparison with that of the cerebral white matter (WM), and this absolute differentiation can be quantified by GWR (14, 15).

## Quantified Analysis of Cerebral Edema After CA With GWR-BG

**Figure 2:**
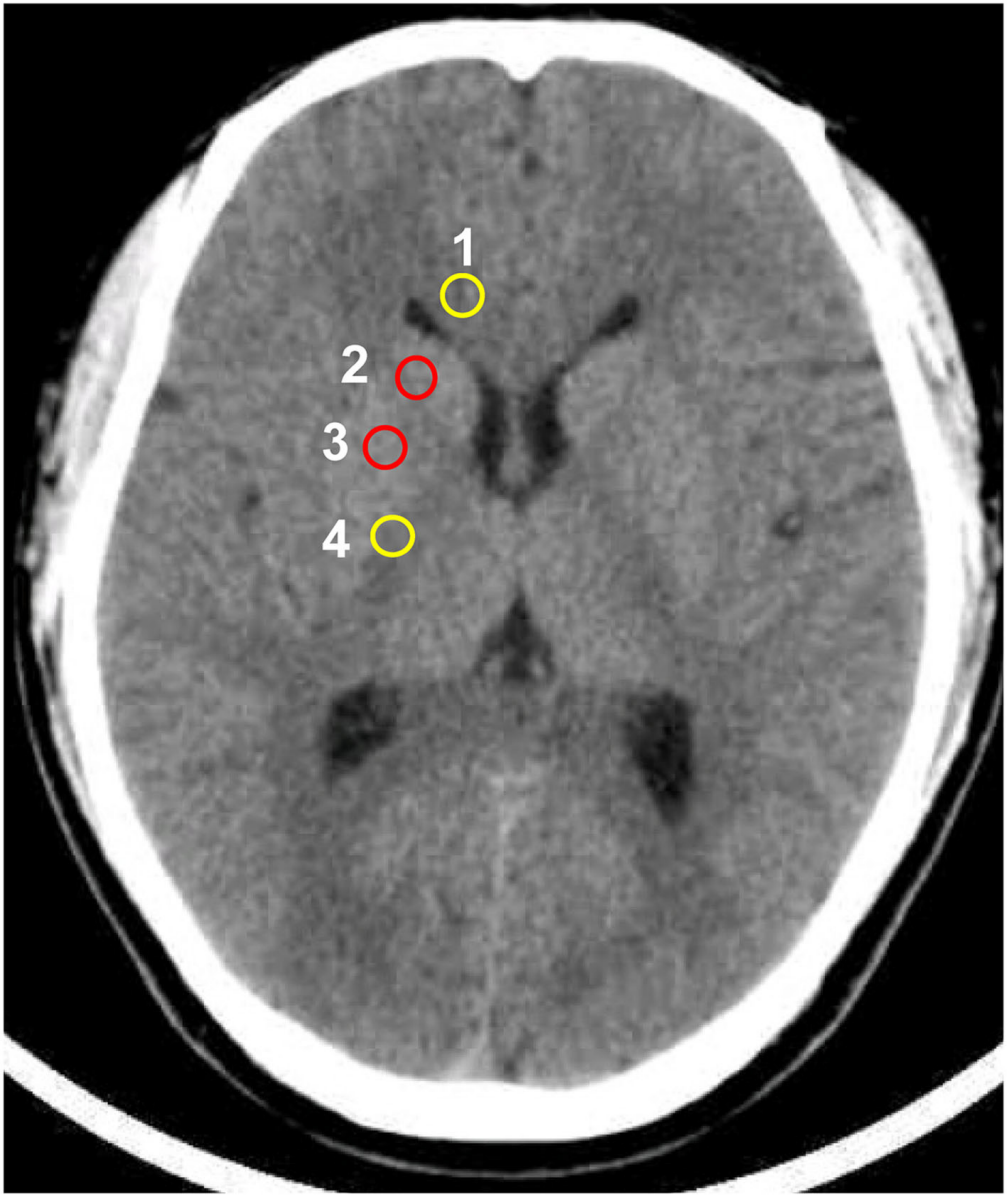
Circular regions of interest drawn bilaterally in the following regions: 1 corpus callosum, 2 caudate nucleus, 3 putamen, 4 posterior limb of the internal capsule.

In the investigational assessment of the GWR-BG, investigators were blinded to patients' outcomes when performing GWR-BG measurements. Circular regions of interest were used to assess the densities of GM and WM in Hounsfield units (HU). GM densities were measured in the putamen (PU) and caudate nucleus (CN), while WM densities were measured in the corpus callosum (CC) and the posterior limb of the internal capsule (PLIC, Figure 2). GWRs can be divided into five subgroups according to their locations: (PU + CN)/(CC + PLIC), CN/PLIC, CN/CC, PU/PLIC, and PU/CC (Table 1). The GWR assessed using (PU + CN)/(CC + PLIC) also referred to as GWR-BG or GWR_basalganglia_. The other subgroups were termed GWR-simple or GWR-SI. Neurologic outcomes of CA survivors were assessed using the Glasgow-Pittsburgh cerebral performance category (CPC) at the time of discharge, and 3 months or 6 months later. A CPC of 1–2 was defined as a “good” outcome, while a CPC of 3–5 was considered a “poor” outcome.

**Table 1:** The Gray-white matter ratio at the basal ganglia (GWR-BG) in different locations.

		**Gray matter**		**White matter**	
		**Putamen**	**Caudate nucleus**	**Corpus callosum**	**Posterior limb of the internal capsule**
BG	(CN + PU)/(PLIC + CC)	**+**	**+**	**+**	**+**
	CN/PLIC	**-**	**+**	**-**	**+**
SI	CN/CC	**-**	**+**	**+**	**-**
	PU/PLIC	**+**	**-**	**-**	**+**
	PU/CC	**+**	**-**	**+**	**-**

*BG, basal ganglia; CC, corpus callosum; CN, caudate nucleus; PLIC, posterior limb of the capsule; PU, putamen*.

## GWR-BG As An Early Neurologic Outcome Predictor In Comatose CA Survivors

A summary of studies evaluating the GWR-BG for predicting neurologic outcomes is displayed in Table 2. Among these studies, there is considerable variation, including in the number of enrolled patients, the median time to CT after ROSC, the selected cut-off values, and the reported sensitivity. The number of included patients ranged from 8 to 346. The median time to CT after ROSC ranged from 16 min to 8.4 h, the reported specificity ranged from 72.4 to 100%, and the reported sensitivity ranged from 3.3 to 88%. Within subgroups, the sensitivity of GWR-BG ranged from 3.3 to 88%, GWR of CN/PLIC ranged from 3.5 to 63%, GWR of CN/CC was 33.3 or 45.45%, GWR of P/PLIC ranged from 3.0 to 76%, and GWR of P/CC ranged from 3.0 to 76%.

**Table 2:** The sensitivity of Gray-white matter ratio at the basal ganglia (GWR-BG) in predicting poor neurologic outcomes in cardiac arrest survivors.

**GWR**	**Author**	**IHCA/OHCA**	**Including patients**	**TTM**	**Time from ROSC to CT**	**Cut-off**	**Sensitivity**	**Specificity**	**Poor outcome**
(CN+PU) / (PLIC+CC)	Wang (8)	-	58	–	<72 h	1.18	50%	87.5%	CPC3-5, discharge
	Kim (10)	OHCA	51	Y	16 min	1.12	3.3%	100%	CPC3-5, discharge
	Lee BK (11)	OHCA	283	Y	–	1.094	3.5%	100%	CPC3-5, discharge
	Lee YH (12)	OHCA	8	–	–	1.24	88%	100%	CPC3-5, discharge
	Scarpino (16)	IHCA+OHCA	183	Y	<24 h	1.21	50.4%	100%	CPC 3-5, at 6 months
	Scarpino (17)	IHCA	346	Y	–	1.21	48.8%	100%	CPC 4-5, discharge
	Lee BK (18)	OHCA	164	Y	125 min	1.17	26.2%	100%	CPC3-5, discharge
	Gentsch (19)	OHCA+IHCA	98	Y	5 h	1.16	44.3%	100%	CPC3-5, discharge or ICU admission
	Hwan (20)	OHCA+IHCA	91	Y	<24 h	1.21	83.8%	100%	CPC3-5, discharge
CN/PLIC	Lee BK (11)	OHCA	283	Y	–	1.094	3.5%	100%	CPC3-5, discharge
	Lee BK (18)	OHCA	164	Y	125 min	1.138	20%	100%	CPC3-5, discharge
	Son (21)	OHCA	58	Y	79 min	NA	12.2%	100%	CPC 3-5, at 3 months
	Jeon (22)	–	39	Y	90 min	1.15	39.4%	100%	CPC3-5, at 6 months
	Choi (23)	OHCA	28	–	3.9 h	1.2	63%	100%	GOS1-2, discharge
	Lee BK (24)	OHCA+IHCA	186	Y	69.5 min	1.1	19.8%	100%	CPC3-5, discharge
CN/CC	Son (21)	OHCA	58	Y	79 min	NA	33.3%	100%	CPC 3-5, at 3 months
	Jeon (22)	–	39	Y	90 min	1.15	45.45%	100%	CPC3-5, at 6 months
PU/PLIC	Wan (8)	–	58	–	<72 h	1.16	50%	93.8%	CPC3-5, discharge
	Lee BK (11)	OHCA	283	Y	–	1.06	3.5%	100%	CPC3-5, discharge
	Lee YH (12)	OHCA	8	–	–	1.21	76%	100%	CPC3-5, discharge
	Lee BK (18)	OHCA	164	Y	123.5 min	1.12	9.7%	100%	CPC3-5, discharge
	Gentsch (19)	OHCA+IHCA	98	Y	5 h	1.16	44.3%	100%	CPC3-5, discharge or ICU admission
	Son (21)	OHCA	58	Y	79 min	NA	3.0%	100%	CPC 3-5, at 3 months
	Hanning (25)	–	84	–	8.4 h	1.251	58.2%	82.8%	CPCP3-5, admission to the ICU or the general ward
PU/CC	Wang (8)	–	58	–	<72 h	1.1	28.6%	100%	CPC3-5, discharge
	Lee BK (11)	OHCA	283	Y	–	1.107	5.6%	100%	CPC3-5, discharge
	Lee BK (18)	OHCA	164	Y	125 min	1.2	43.5%	100%	CPC3-5, discharge
	Son (21)	OHCA	58	Y	79 min	NA	6.06%	100%	CPC 3-5, at 3 months
	Jeon (22)	-	39	Y	90 min	1.1	30.3%	100%	CPC3-5, at 6 months
	Lee BK (24)	OHCA+IHCA	186	Y	69.5 min	1.17	52.9%	100%	CPC3-5, discharge

*IHCA, in-hospital cardiac arrest; OHCA, out-of-hospital cardiac arrest; BG, basal ganglia; CC, corpus callosum; CN, caudate nucleus; CPC, Cerebral Performance Category score; PLIC, posterior limb of the capsule; PU, putamen*.

## Factors Affecting the Sensitivity of GWR-BG In Predicting Poor Neurologic Outcomes

According to the above literature review, it is obvious that the specificity of GWR-BG in predicting poor neurologic outcomes following CA is high. However, the sensitivity of this method was found to vary considerably in the previous studies. These differences can be explained by numerous factors, including differences in definitions of poor outcome, regions of interest (ROIs) for determining the GWR, cut-off values, etiologies, time from ROSC to CT, investigators, and CT scanners. Previously, a study evaluating CA conducted in dogs demonstrated that the asphyxiation group showed worse ischemic anoxic brain damage than that of the ventricular fibrillation group (26). Similarly, a retrospective study revealed that the sensitivity of GWR-BG and GWR of P/CC was higher in predicting the outcome of hypoxic CA survivors than that of non-hypoxic CA survivors (18). Post-CA hypoxic-ischemic brain injury is a dynamic process during which the HU of the GM decreases more quickly than that of the WM. Therefore, GWR measurements display a declining trend in a time-dependent manner (27). A recent study reported that the average first GWR-BG measurement was 1.186 ± 0.07 in poor outcome CA survivors, but this value decreased to 1.088 ± 0.143 when cranial CT was performed again during the following seven days (28). According to the median time duration from ROSC to CT, CA survivors were divided into a ≤ 26 min group, a 26–50 min group, a 50–107 min group, and a >107 min group (11). Lee BK et al. (11) found that the performances of the GWR-BG, GWR of CC/PLIC, PU/CC, and PU/PLIC were not significantly different in predicting poor outcomes. On the contrary, Wang et al. (8) reported that GWR-BG and GWR of PU/PLIC values determined using early (24 h) and 24 h later (24–72 h) cranial CTs were significantly different between measurements. In contrast to the two aforementioned studies, Streitberger et al. (29) demonstrated that the sensitivity of the 0–6 h cranial CT group and the 6–24 h cranial CT group were 20% and 10%, respectively, while that of the >24 h group was 41% in predicting poor outcomes post CA using the same GWR cut-off value and specificity.

Interestingly, another study indicated that the HU values of the GM and WM in the normal adult brain varied statistically significantly according to the implemented CT scanner (the Somatom Definition, Lightspeed VCT vs. the Discovery CT750 HD CT). The HU values of the caudate nucleus, putamen, corpus callosum, and posterior limb of the internal capsule, as well as GWR-BG, GWR of PU/PLIC, and CN/CC detected by the Somatom Definition scanner were higher than those detected by the other two CT scanners (30). Finally, though investigators were blinded to patients' clinical outcomes, the sensitivity of the GWR for predicting patient outcomes differed despite using the same GWR cut-off values to assess the same CA survivors (31). Cristia et al. (31) showed that a GWR cut-off of 1.2 yielded difference of sensitivity greater than a cut-off value of 1.1 in predicting poor outcomes in CA survivors.

## Comparison of GWR-Average With GWR-BG In Predicting Poor Outcomes

Instead of using the 16 ROIs required to compute the GWR-average, GWR-BG and GWR-SI only requires obtaining CT measurements from eight or four cranial regions, respectively. Thus, these metrics seem simpler and less time-consuming than the GWR-average. However, some researchers are concerned that the predictive accuracy of these metrics may be correspondingly reduced. This result is still uncertain (Table 3). Wang et al. (8) and Kim et al. (10) demonstrated that the sensitivity of the GWR-average was higher than that of the GWR-BG. On the contrary, Gentsch et al. (19) and Lee et al. (12) showed that the sensitivity of the GWR-BG was higher than that of the GWR-average. Interestingly, Lee et al. (18) showed that GWR of PU/CC displayed the highest sensitivity among GWR-average, GWR-BG, CN/PLIC, PU/CC, and PU/PLIC. A meta-analysis indicated that the GWR-BG presented the highest pooled diagnosis odds ratio for predicting poor outcomes in relation to GWR-average and GWR of PU/PLIC in in-hospital and out-of-hospital CA survivors (32).

**Table 3:** Comparison of the sensitivity between the average gray-white matter ratio (GWR-average) and the gray-white matter ratio at the basal ganglia (GWR-BG) in predicting poor neurologic outcomes in cardiac arrest survivors.

**Author**	**Global brain**		**Basal ganglia**				**Conclusion**
	**Cut-off**	**Sensitivity**	**Cut-off (BG)**	**Sensitivity (BG)**	**Cut-off (SI)**	**Sensitivity (SI)**	
Wang (8)	1.14	38.1%	1.12	28.6%	1.23 (PU/PLIC)	28.6%	The sensitivity of the GWR-average was higher than that of the GWR-BG.
Kim (10)	1.14	13.3%	1.12	3.3%	-	-	
Lee YH (12)	1.23	76%	1.24	88%	1.21 (PU/PLIC)	76%	The sensitivity of GWR-BG was higher than that of the GWR-average.
Lee BK (18)	1.22	28.3%	1.17	26.2%	1.2 (PU/CC)	43.5%	The sensitivity of the GWR with respect PU/CC was the highest of the evaluated measures.
Gentsch (19)	1.61	39.3%	1.16	44.3%	1.11 (PU/PLIC)	44.3%	The sensitivity of the GWR-SI was the same as that of the GWR-BG, and was better than that of the GWR-average
Lee BK (11)	1.13	3.5%	1.1	3.5%	1.107 (PU/PLIC)	3.5%	No statistically significant difference was found.

*BG, basal ganglia; CC, corpus callosum; PLIC, posterior limb of the capsule; PU, putamen*.

## Increasing Sensitivity by Combining the GWR-BG With Other Prognostic Tests

As described before, clinical examinations, MRI, EEG, and serum biomarker analysis are useful for neuroprognostication (5, 33). Neurological examination is convenient for emergency physicians. However, the ocular reflex, the corneal reflex, and the GCS-M (the motor response components of the Glasgow Coma Scale) are affected by sedatives. MRI can be implemented early to detect and quantify cerebral edema caused by ischemic hypoxic brain injury. However, it is not suitable for unstable patients or patients treated with ECMO (34). Moreover, EEG results are easily affected by sedatives and require continuous monitoring and interpretation by medical professionals. Serum biomarkers, such as NSE (neuron-specific enolase) and S-100B, are not affected by sedatives and can be determined from easily collected samples. However, patients with traumatic brain injury may show false positive results, and the sensitivity of these tests is associated with patients' age, the detection threshold, the detection time, and the employed methodology (33, 34). Though several researchers have improved the test sensitivity of this method through a variety of approaches, such as withdrawing sedatives and using a quantitative pupillometer, some methods are not suitable for critically ill patients (Table 4).

**Table 4:** The comparative advantages and disadvantages of common examinations and tests predicting neurologic outcomes.

**Methods**	**Advantage**	**Disadvantage**	**Improved methods**
Absent pupillay light response	Clinical examinations are simple, convenient, and do not require specialized equipment.	The false positive rate is approximately 33%, and the reliability of the findings are influenced by sedatives (6).	Assessment is performed by automated infrared pupillometry without sedatives 72 h after cardiac arrest (35, 36).
Absent corneal reflex		The false positive rate is approximately 4%, and the reliability of the findings are influenced by neuromuscular blocking drugs (37).	Stopping sedatives.
GCS-M		This method is prone to interference from residual effects of sedatives or muscle relaxants.	Stopping sedatives and muscle relaxants (37).
Myoclonus		Myoclonus is difficult to distinguish from status epilepticus and is also influenced by sedatives and muscle relaxants.	Stopping muscle relaxants and sedatives.
GWR (CT)	Test performance is relatively simple, and the results are stable, not influenced by sedatives, and exclude cerebral hemorrhage.	The reliability of this method is affected by the CA-CT performance time, the measurement area, the selected cut-off values, and inter-observer variability.	It is advised to perform cranial CT at 48 h following ROSC (14), along with unifying measurement regions and GWR cut-off values.
ADC (MRI)	MRI can quantify the degree of cerebral edema with high sensitivity and is not easily influenced by sedatives.	MRI is not suitable for unstable cardiac arrest survivors, and the associated sensitivity is influenced by detection time, measurement region(s), and cut-off values.	It is advised to perform MRI 5 days after ROSC (38).
EEG	Continuous monitoring	The result is influenced by sedatives and requires professional interpretation.	Stopping sedatives and continuous monitoring (39).
NSE, S-100B	The biosamples are easily collected, continuous detection is possible, and the findings are not influenced by sedatives.	The result represents an assessment dynamic change, and the reliability of this is associated with age, cut-off values, the extent and characteristics of the evaluated brain injury, detection time, and equipment (40–42).	Continuous detection.

*CA, cardiac arrest; CT, computed tomography; ADC, apparent diffusion coefficient; EEG, electroencephalography; GCS-M, GCS-M (the motor response components of the Glasgow Coma Scale); GWR, gray-white matter ratio; MRI, magnetic resonance imaging; NSE, neuron-specific enolase; ROSC, return of spontaneous circulation*.

A multimodal approach is recommended to improve GWR reliability through implementing evidence-based guidelines. The predictive accuracy of GWR is increased through combinations with other prognostic tests. Chae et al. (43) found that the sensitivity of GWR alone was only 5.5% in predicting poor neurologic outcomes, whereas the sensitivity increased to 27% when combined with optic nerve sheath diameter (ONSD) measurements. Similarly, Hwan et al. (20) reported that the sensitivity of the GWR combined with ONSD measurements was 92%, which was superior to that of the GWR alone. The sensitivity of the GWR with respect to the PU/CC was 27.3, and this increased to 78.6% after being combined with ONSD measurements in terms of predicting poor neurologic outcomes. Combined GWR and MRI evaluations also resulted in similar increases in sensitivity in prior research. For example, the sensitivity of a putamen/CC assessment combined with MRI findings led to an increase from 30.3 to 81.8% within a prior study (22). Moreover, both Youn et al. (44) and Roman et al. (45) confirmed that combining GWR assessments with EEG or SSEP evaluations was superior to any individual test in terms of predicting mortality and poor neurologic outcomes. Consistent with these findings, Wu et al. (46) reported that the sensitivity of PU/PLIC alone was 8% in evaluating poor neurologic outcomes but increased to 50% when combined with an absent pupillary light response assessment.

## Perspectives

Post-cardiac arrest syndrome (PCAS) is a complex and critical complication occurring in resuscitated patients who have undergone cardiac arrest. This syndrome includes post-cardiac arrest brain injury, post-cardiac arrest myocardial dysfunction, and a systemic ischemia/reperfusion response (47). Post-CA ischemic hypoxic brain injury is the crucial organ damage associated with neuronal death and cerebral edema. Early post-CA cranial CT scans display effacement of brainstem cisterns and cerebral sulci, decreased cortical density, and loss of the normal differentiation of the GM and the WM. The latter CT finding has also been termed the “reverse sign” or “loss of boundary” in research conducted over the course of the past four decades (48). During this time, the reverse sign has frequently been associated with poor prognoses in CA patients (49). Inamasu et al. (50) visually assessed post-CA prognoses with respect to asphyxia by examining loss of gray-white matter discrimination at the basal ganglia. The sensitivity of this method was 100% for predicting death. In a later effort, Torbey et al. (7) quantitatively analyzed GWR values and found that a GWR-BG of less than 1.18 predicted death with near certainty.

Increasing evidence has shown that a decreased GWR-BG strongly predicts poor neurologic outcomes in the years following CA. However, the reliability of this method is still vigorously debated. Previous analyses have determined that the sensitivity of the GWR-BG is affected by a variety of factors, including differences in the definition of poor neurologic outcomes, the time from ROSC to CT, the GWR measurement regions, and the GWR cut-off values. In addition to CPC scores, Modified Rankin Scale (mRS) and Glasgow Outcome Scale (GOS) scores have been used to define neurological outcomes in some prior studies (23, 27, 46). Neurological outcomes are typically assessed during the hospital discharge process. However, a few studies have followed up on patients for up to 3–6 months following hospital discharge (21, 22). The median time from ROSC to CT has also varied significantly, ranging from 16 min to 8.4 h. We further reviewed studies with GWR-BG sensitivities below <20% and found that the time from ROSC to CT was far less than that recommended by medical guidelines (10, 18, 21, 24). Compared with GWR-average, the GWR-BG and especially the GWR-SI have been found to reduce the required scope of the GM and WM measurement regions and are quite simple to measure. However, the factors affecting the sensitivity of these tests remain unclear. The sensitivity of the GWR has been associated with inter-observer variability (31). Moreover, automated GWR determination allows for the observation of global GWR values as well as the isolation of anatomical locations based on cranial CT densitometric information. This methodology seems more precise and objective than manual determination and the sensitivity of the GWR calculated by this automatic analysis methodology can reach up to 92.7% (51).

In conclusion, there are many factors affecting the predictive accuracy of GWR-BG. Sensitivity may significantly increase after standardizing the ROSC to the CT interval, unifying evaluation criteria, procedural protocols, and cut-off values, and combining the GWR-BG with data from various neurological examinations, EEG, MRI, and biomarker assessments.

## Author Contributions

FZ and HW conceived the study, searched literature, and wrote the manuscript. MJ and ZW checked and classified the literature. HD and YH was responsible for drawing extracting data and drawing. LG and YC conceived and supervised the study, interpreted clinical data, and revised the manuscript. All authors have accepted responsibility for the entire content of this submitted manuscript and approved submission.

## Funding

This present work was supported by a grant from the National Natural Science Foundation of China (Grant Nos. 81471836 and 81772037 to YC and Grant No. 81801883 to YH).

## Conflict of Interest

The authors declare that the research was conducted in the absence of any commercial or financial relationships that could be construed as a potential conflict of interest.

## Publisher's Note

All claims expressed in this article are solely those of the authors and do not necessarily represent those of their affiliated organizations, or those of the publisher, the editors and the reviewers. Any product that may be evaluated in this article, or claim that may be made by its manufacturer, is not guaranteed or endorsed by the publisher.
